# Imaging of Metastatic Cancer Cells in Sentinel Lymph Nodes using Affibody Probes and Possibility of a Theranostic Approach

**DOI:** 10.3390/ijms20020427

**Published:** 2019-01-19

**Authors:** Makoto Tsuchimochi, Haruka Yamaguchi, Kazuhide Hayama, Yasuo Okada, Tomoyuki Kawase, Takamasa Suzuki, Norio Tsubokawa, Noriaki Wada, Atsushi Ochiai, Satoshi Fujii, Hirofumi Fujii

**Affiliations:** 1 Emeritus Professor, The Nippon Dental University, Tokyo, Japan, formerly of the Department of Oral and Maxillofacial Radiology, The Nippon Dental University School of Life Dentistry at Niigata, 1-8 Hamaura-cho, Chuo-ku, Niigata 951-8580, Japan; 2 Department of Life Science Dentistry, The Nippon Dental University, 1-9-20 Fujimi, Chiyoda-ku, Tokyo 102-8159, Japan; harukay@ngt.ndu.ac.jp; 3 Department of Oral and Maxillofacial Radiology, The Nippon Dental University School of Life Dentistry at Niigata, Niigata 951-8580, Japan; hayama@ngt.ndu.ac.jp; 4 Basil Hetzel Institute for Translational Health Research, The Queen Elizabeth Hospital, 28 Woodville Road Woodville South, SA 5011, Australia; 5 Department of Pathology, The Nippon Dental University School of Life Dentistry at Niigata, Niigata 951-8580, Japan; yokada@ngt.ndu.ac.jp; 6 Division of Oral Bioengineering, Institute of Medicine and Dentistry, Niigata University, Division of Oral Bioengineering, Department of Tissue Regeneration and Reconstitution, Niigata University Graduate School of Medical and Dental Sciences, Niigata 951-8541, Japan; kawase@dent.niigata-u.ac.jp; 7 Department of Electrical and Electronic Engineering, Faculty of Engineering, Niigata University, Niigata 950-2181, Japan; takamasa@eng.niigata-u.ac.jp; 8 Faculty of Engineering, Niigata University, Niigata 950-2181, Japan; ntsuboka@eng.niigata-u.ac.jp; 9 Department of General Surgery, Tokyo Dental College Ichikawa General Hospital, Ichikawa, Chiba 272-8513, Japan; nowada@tdc.ac.jp; 10 Division of Biomarker Discovery, Exploratory Oncology Research and Clinical Trial Center, National Cancer Center, Kashiwa, Chiba 277-8577, Japan; aochiai@east.ncc.go.jp; 11 Laboratory of Cancer Biology, Department of Integrated Biosciences, Graduate School of Frontier Sciences, University of Tokyo, Kashiwa, Chiba 277-8561, Japan; 12 Division of Pathology, Exploratory Oncology Research & Clinical Trial Center, National Cancer Center, Kashiwa, Chiba 277-8577, Japan; sfujii@east.ncc.go.jp; 13 Division of Functional Imaging, Exploratory Oncology Research and Clinical Trial Center, National Cancer Center, Kashiwa, Chiba 277-8577, Japan; hifujii@east.ncc.go.jp

**Keywords:** sentinel lymph node, molecular imaging, affibody, EGFR, HER2, near-infrared fluorescence, scintigraphy, image-guided surgery, photoimmunotherapy, theranostics

## Abstract

The accurate detection of lymph node metastases is essential for treatment success in early-stage malignant cancer. Sentinel lymph node (SLN) biopsy is the most effective procedure for detecting small or micrometastases that are undetectable by conventional imaging modalities. To demonstrate a new approach for developing a more efficient SLN biopsy procedure, we reported a two-stage imaging method combining lymphoscintigraphy and near-infrared (NIR) fluorescence imaging to depict metastatic cancer cells in SLNs in vivo. Furthermore, the theranostic potential of the combined procedure was examined by cell culture and xenograft mouse model. Anti-HER2 and anti-epidermal growth factor receptor (EGFR) affibody probes were used for NIR fluorescence imaging. Strong NIR fluorescence signal intensity of the anti-EGFR affibody probe was observed in SAS cells (EGFR positive). Radioactivity in the SLNs was clearly observed in the in vivo studies. High anti-EGFR affibody NIR fluorescence intensity was observed in the metastatic lymph nodes in mice. The addition of the IR700-conjugated anti-EGFR affibody to the culture medium decreased the proliferation of SAS cells. Decreased proliferation was shown in Ki-67 immunohistochemistry in xenograft tumors. Our data suggest that a two-stage combined imaging method using lymphoscintigraphy and affibody probes may offer the direct visualization of metastatic lymph nodes as an easily applied technique in SLN biopsy. Although further animal studies are required to assess the effect of treating lymphatic metastasis in this approach, our study results provide a foundation for the further development of this promising imaging and treatment strategy for earlier lymph node metastasis detection and treatment.

## 1. Introduction

Lymph node staging is a significant prognostic factor in many malignancies, especially solid malignant tumors. The accurate detection of lymph node metastases is essential for treatment success in early-stage malignant cancer. Sentinel lymph node (SLN) biopsy is the most effective procedure for detecting small or micrometastases that are undetectable by conventional imaging modalities, such as computed tomography (CT), magnetic resonance imaging (MRI), or positron emission tomography (PET). SLNs are the first nodes in the regional lymph basin to direct drain lymph from primary lesions. Histopathological examination of one or a few SLNs can reveal the stage of the regional lymph drainage field. SLN biopsy has been a standard procedure in breast cancer and melanoma and has since been introduced in head and neck cancer and other malignancies [1,2,3,4]. SLN biopsy is a minimally invasive procedure. Patients with a negative SLN biopsy are spared unnecessary extensive surgery, thereby reducing the risk of postsurgical complications, such as lymphedema and numbness, while those with a positive SLN biopsy undergo the lymph node dissection surgery at an early stage.

A combination of ^99m^Tc radiopharmaceuticals and blue dye is generally used for SLN biopsy procedures. In searching for the radioactive lymph nodes, the hand-held gamma probe is utilized [1,2]. The small gamma camera was developed to facilitate better identification of the radioactive lymph nodes in the operating theater, and some are now commercially available [5,6]. Near-infrared (NIR) fluorescence imaging using indocyanine green (ICG) has also been introduced for the identification of SLNs. The invisible fluorescence signals can be displayed on a screen using a portable fluorescence camera. This method improves the sensitivity of detecting SLNs because ICG accumulates and remains in lymph nodes longer than visible dyes, which rapidly flow out from vessels and lymph nodes; additionally, ICG NIR fluorescence can be detected in deep tissues. Recently, combined agents for both NIR fluorescence imaging and radionuclide imaging have been developed and used in SLN biopsy. This combination allows surgeons to find deeply located SLNs via the better tissue penetration of gamma rays and to detect clear SLN fluorescence with normal anatomy [7,8]. A novel radiopharmaceutical was recently approved by the FDA and European Medicines Agency; ^99m^Tc-Tilmanocept targets the CD206 mannose-binding receptors expressed on nodal reticuloendothelial cells for SLN detection [8,9]. Although these developed procedures and methods help to identify SLNs, they still cannot enable the imaging of cancer cells, per se, inside lymph nodes.

Several studies aiming to detect lymph node micrometastases in vivo during procedures are currently underway. Dual-modality imaging probes ^124^I-cRGDY-PEG-C dots (cRGDY: cyclic arginine-glycine-aspartic acid-tyrosine; PEG: methoxy-terminated polyethylene glycol; C dots: fluorescent core-shell silica nanoparticles) were used for detecting SLN metastases in a mouse study. These imaging probes contained peptide ligands and exhibited high-affinity/avidity binding to αvβ3 integrin-expressing melanoma xenografts in mice [10]. The high sensitivity to nodal metastases, real-time imaging of lymphatic drainage patterns, and favorable blood clearance rate of the imaging probes led to a clinical trial of their capability in the specific determination of tumor-bearing lymph nodes [11,12,13]. Animal studies using antibodies were also conducted for the real-time imaging of lymph node metastases [14,15]. The NIR imaging probe cetuximab, an antibody against epidermal growth factor receptor (EGFR), and trastuzumab, an antibody against human epidermal growth factor receptor 2 (HER2), were labeled with a fluorophore (IRDye 800) and studied to determine their ability to detect tumor metastases within SLNs in a mouse model. These imaging probes could distinguish metastatic lymph nodes from nonmetastatic lymph nodes [15]. Recently, photoacoustic imaging, also referred to as optoacoustic imaging, was introduced for biomedical imaging. The approach combines optical imaging with ultrasound imaging. Nonionizing laser pulses are delivered into biological tissues. The absorbed energy is converted into a local temperature increase, and the thermal expansion generates ultrasound waves. This signal is dependent on the endogenous molecules, and the contrast medium is detected by an ultrasonic transducer for real-time imaging [16]. This new imaging modality is used in preclinical settings for not only localizing SLNs but also detecting metastases in lymph nodes [17,18,19].

We used an affibody molecule, which is an antibody mimetic, instead of using an antibody to target HER2 or EGFR in metastatic cancer cells. This engineered affinity protein is synthesized based on the immunoglobulin-binding region of staphylococcal protein A to recognize various molecules, such as proteins or peptides [20,21,22]. Preclinical or clinical applications of affibodies for diagnosis and therapy were reported [23,24,25,26,27].

In our animal studies, we found that an affibody combination probe could image metastatic cancer cells in SLNs with a rational approach for depicting the lesion. Deeply localized SLNs could easily be explored by ^99m^Tc-phytate scintigraphy, and affibody NIR imaging could identify metastatic cancer cells in the lymph nodes in real time during the biopsy procedure. Furthermore, the theranostic possibility of imaging metastatic cancer cells and treating them with photoimmunotherapy (PIT) was shown.

## 2. Results

### 2.1. Cell Culture

**Figure 1:**
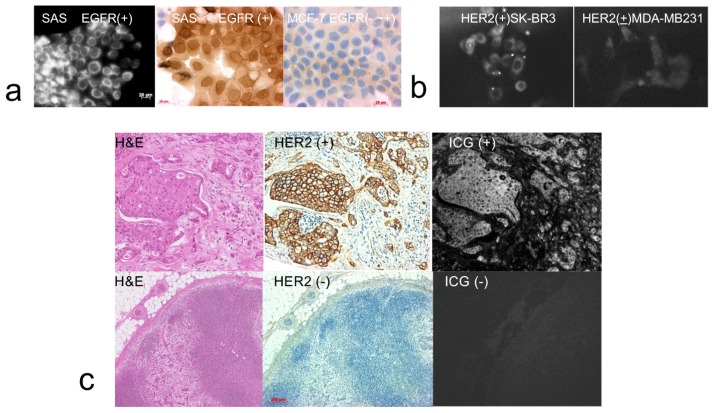
Near-infrared (NIR) imaging of cell lines by the addition of affibody probes to conditioned medium (**a**,**b**). SAS cells showed strong fluorescence signals of anti-EGFR affibody imaging probes (**a**, left). SAS cells (**a**, middle) expressed higher anti-epidermal growth factor receptor (EGFR) levels than MCF-7 cells (**a**, right). SK-BR3 cells showed stronger fluorescence signals in anti-HER2 affibody imaging probes than MDA-MB231 cells (**b**). Histological section study (**c**). Anti-HER2 affibody probe was administered to histological sections of lymph nodes from breast cancer patients. Metastatic cancer cells are shown after hematoxylin and eosin (H&E) staining (**c**, upper row, left). Human epidermal growth factor receptor 2 (HER2) expression was positive in metastatic cancer cells by immunohistochemical staining (**c**, upper row, middle). High-intensity NIR signals from the probe identically corresponded with the area of increased HER2 expression (**c**, upper row, right). In the HER2-negative metastatic lymph node section (**c**, lower row), immunohistochemical staining for HER2 and NIR signals were not visible in metastatic cancer cells (**c**, lower row, middle, right). Scale bar in (**a**): 20 μm. Scale bar in (**c**): 200 μm.

To verify the NIR imaging efficacy of the anti-EGFR affibody probe, the anti-EGFR affibody probe was added to the conditioned medium of SAS cells or MCF-7 cells. Strong NIR fluorescence from the anti-EGFR affibody probe was observed in the SAS cells (positive EGFR expression cell line) in contrast to the negative signal observed from the MCF-7 cells (negative EGFR expression cell line). The EGFR expression status of the cells was confirmed by immunohistochemistry. SAS cells showed positive expression (Figure 1a). SK-BR3 cells showed stronger fluorescence signals in anti-HER2 affibody imaging probes than MDA-MB231 cells (Figure 1b).

### 2.2. Section Analysis

The NIR imaging efficacy of the anti-HER2 affibody probe was then examined. The deparaffinized histological sections of 11 lymph nodes (four sections each) from four patients with breast cancer metastasis were used. HER2 expression was found in nine lymph nodes and not in two lymph nodes by immunohistochemistry. High NIR fluorescence from the anti-HER2 affibody probe was located precisely in the regions identified as HER2-positive areas by immunohistochemical staining in the nine HER2-positive lymph nodes. In the two HER2-negative lymph nodes, NIR fluorescence was not observed. HER2-positive and HER2-negative control slides were also studied. We confirmed the presence of NIR fluorescence in the HER2-positive slide and not in the HER2-negative side (Figure 1c).

### 2.3. Combined Imaging

**Figure 2:**
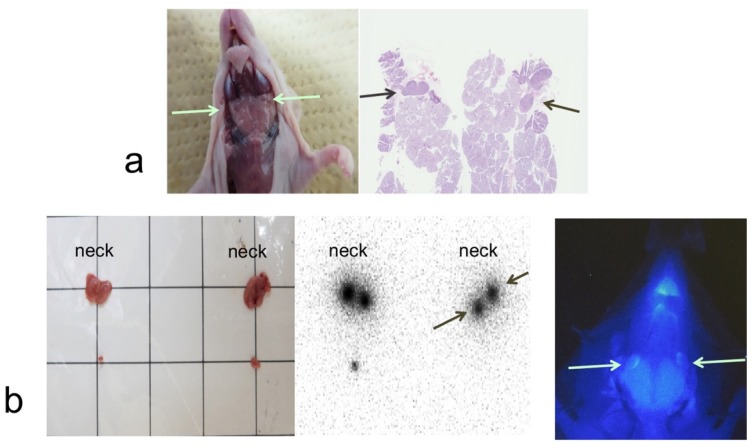
Neck lymph node in normal mouse (**a**). The arrows indicate neck lymph nodes in dissection. H&E staining showed lymph nodes in the excised cervical tissue (**a**). Two-phase combined imaging of SLNs in normal mice (**b**). ^99m^Tc-Phytate and anti-EGFR affibody probe were injected into the tongue of mice. Two excised neck specimens with lymph nodes from two mice are shown (**b**, left). Autoradiography showed that ^99m^Tc radioactivity accumulated in the lymph nodes of each neck specimen and one solitary lymph node in the corresponding tissues of the left panel (**b**, middle). NIR fluorescence was localized in the lymph nodes after anti-EGFR affibody probe injection into the tongue (**b**, right).

A combined imaging study was performed in nine mice to examine whether both imaging agents drain to lymph nodes. Increased radioactivity and high NIR signal intensity in the lymph nodes were found in the neck of all nine mice at one hour after ^99m^Tc-phytate injection. Among all 21 excised lymph nodes, a high uptake of ^99m^Tc was observed in 19 lymph nodes, and a high NIR signal intensity was observed in 20 lymph nodes. Increased radioactivity and a high NIR signal intensity were found in the same 19 out of 21 lymph nodes (Figure 2a,b).

### 2.4. NIR Fluorescence Imaging of Cancer Cells in Lymph Nodes

**Figure 3:**
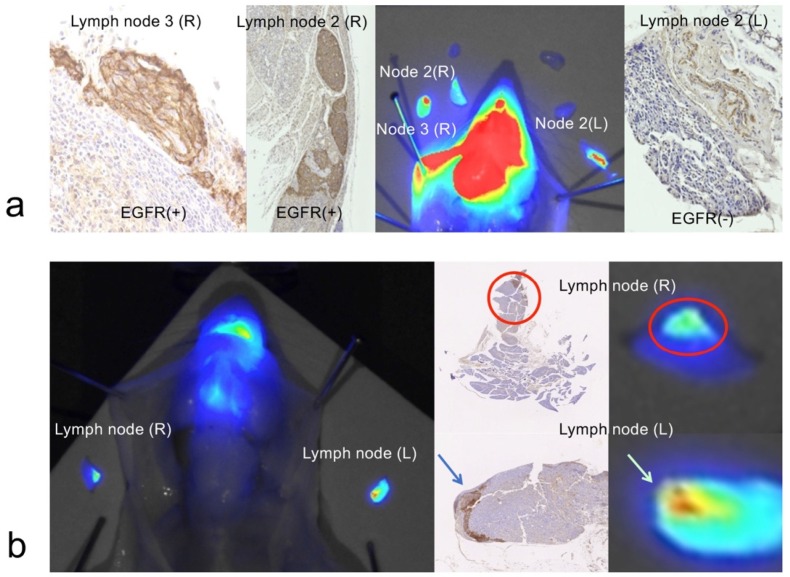
Imaging of metastatic cancer cells in lymph nodes. (**a**) In a mouse lymph node metastasis model, an anti-EGFR affibody probe was injected into the mouse tongue 24 h prior to sacrifice and lymph node dissection. Six lymph nodes were excised; three lymph nodes were highly fluorescent, and the remaining three lymph nodes were not fluorescent. Immunohistochemical staining for EGFR was found in fluorescence-positive lymph nodes (lymph node 2 (R), lymph node 3 (R)). EGFR expression was not visible in the nonfluorescent lymph node (lymph node 2 (L)). (**b**) Two lymph nodes, one from each side of the mouse, were dissected 24 h after anti-EGFR affibody probe injection into the tongue. The indocyanine green (ICG) fluorescence signal was obvious and corresponded to the immunohistochemically stained EGFR expression in the lymph nodes (red circles and arrows). The panel on the right is a magnified view of the two lymph nodes in the left panel of the NIR images.

Twenty-four hours after injection of the anti-EGFR affibody probe, 14 lymph nodes were excised from four mice and examined. NIR fluorescence signals and EGFR expression were analyzed in all 14 lymph nodes by NIR imaging experiments and immunohistochemical studies. High NIR fluorescence was found in all eight EGFR-positive lymph nodes. There were weak or no fluorescence signals in all six EGFR-negative lymph nodes. A high NIR fluorescence signal in the lymph nodes represented lymph node metastasis (Figure 3a,b).

### 2.5. Dynamic NIR Fluorescence Imaging

**Figure 4:**
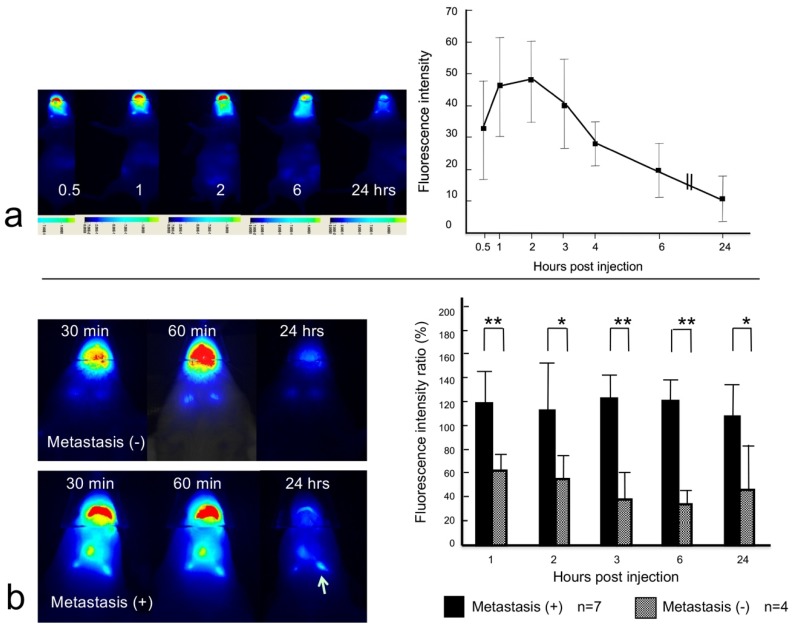
Dynamic imaging study. (**a**) NIR images showed changes in the signal from the anti-EGFR affibody probe in the lymph nodes of a normal control mouse (left). The fluorescence signal intensity was examined at 0.5, 1, 2, 3, 4, 6, and 24 h after the tongue injection in two mice (total four lymph nodes). The peak intensity was observed at one and two hours after the injection (right). The error bar shows the standard deviation. (**b**) The left panels are images of metastatic and nonmetastatic lymph nodes. Weak, almost equal NIR signal intensity was found in two nonmetastatic lymph nodes at 0.5 and 1 h post-injection. The signal intensity almost disappeared at 24 h after the injection (top, left). A high signal intensity remained in the metastatic lymph node (arrow) at 24 h after injection (bottom, left). The time-dependent NIR signal intensity of the anti-EGFR affibody probe is represented as a percentage of the initial signal intensity (30 min). The signal intensity ratio was greater for metastatic lymph nodes than nonmetastatic lymph nodes at 1, 2, 3, and 6 h after the injection (right panel). * *p* < 0.05; ** *p* < 0.01. The error bar shows the standard deviation.

The time dependence of the NIR signal intensity in the lymph nodes (total four lymph nodes) was examined (0.5, 1, 2, 3, 4, 6, and 24 h after injection) in two normal control mice. The peak intensity was observed at one hour and two hours after injection (Figure 4a). Images were also captured in a lymph node metastasis mouse model at 0.5, 1, 2, 3, 6, and 24 h after injection of the anti-EGFR affibody probe in 11 lymph nodes from three mice. The NIR signal intensity in the lymph nodes was presented as a percentage of the fluorescence intensity at baseline, i.e., 30 min after the injection. The signal intensity ratio was higher in metastatic lymph nodes (*n* = 7) than in nonmetastatic lymph nodes (*n* = 4) at 1, 2, 3, and 6 h after the injection. The signal intensity ratio was significantly higher in metastatic lymph nodes than in nonmetastatic lymph nodes at three hours (122.6 ± 21.8 for metastatic lymph nodes vs. 36.8 ± 22.3 nonmetastatic lymph nodes, *p* < 0.01) and six hours (121.6 ± 18.2 for metastatic lymph nodes vs. 33.1 ± 12.8 for nonmetastatic lymph nodes, *p* < 0.01) after injection of the anti-EGFR affibody probe (Figure 4b).

### 2.6. In Vitro PIT

**Figure 5:**
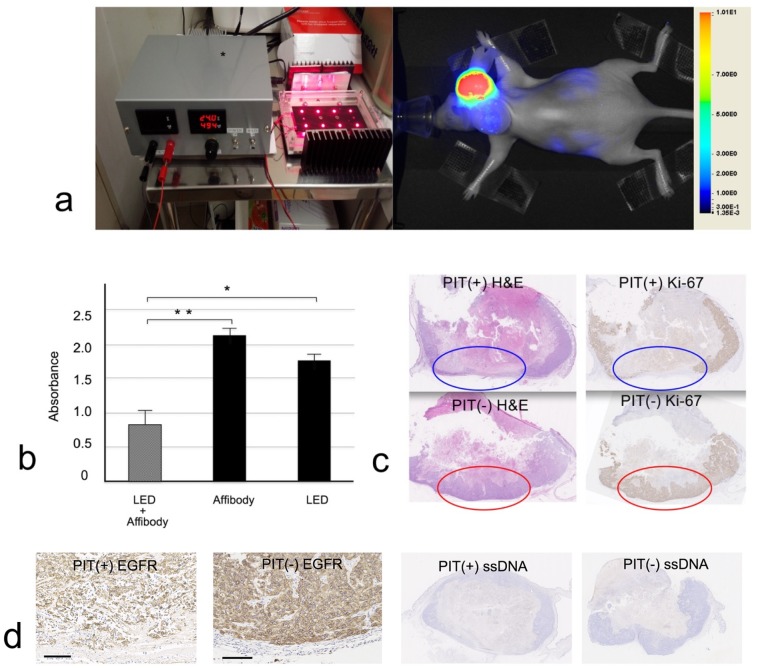
(**a**) Image of a custom-built illuminator (light emitting diode (LED) emission: peak wavelength, 690 nm). Image of SAS xenograft tumor in the back of a mouse after anti-EGFR affibody photoimmunotherapy (PIT). The image was captured one hour after the probe was injected into the right tumor. The contralateral xenograft tumor served as a control (right). (**b**) The CCK-8 assay showed that the combination of the EGFR affibody IR700 probe and NIR irradiation decreased the survival rate of SAS cells rather than exposure to NIR or the EGFR affibody IR700 probe alone (*p* < 0.0001). LED, NIR irradiation (**c**) decreased proliferation in the bottom region of an SAS xenograft tumor treated with PIT, as determined by immunohistochemical staining for Ki-67 (top row), compared to that in a nontreated xenograft tumor (bottom row) (panoramic digital-image source lens, ×40). Circles show the bottom region of tumors (blue = PIT-treated, red = PIT-untreated). (**d**) Immunohistochemical staining for EGFR was performed. Tumor cells were necrotic in the bottom region of the PIT-treated tumors. In the corresponding region of PIT-untreated tumor, EGFR-expressing tumor cells were proliferating. ssDNA immunohistochemistry, which was used for assessing apoptosis, showed negative staining in both PIT-treated and PIT-untreated tumors (panoramic digital-image source lens, ×40, scale bar: 100 μm).

The CCK-8 assay results show that the combined administration of affibody PIT probes and NIR radiation (0.86 ± 0.18) decreased the survival rate of SAS cells compared with exposure to NIR radiation (1.77 ± 0.14) or the EGFR affibody PIT probe (2.12 ± 0.10) alone (*p* < 0.0001) (Figure 5a,b). The mean value of the light emitting diode (LED) solely radiated occasion was lower than the value of the affibody alone condition, though there were no significant differences. The reason for the decrease may have been heat produced by LED light. Even LED lights radiate heat, and the heat might have influenced the cell viability.

### 2.7. In Vivo PIT

Twelve SAS xenograft tumors (three mice) were used for the PIT experiment. Six tumors were irradiated by the custom-built NIR illuminator at 200 mW/cm^2^ and 500 mA. The other six contralateral tumors were not exposed to NIR light as a control. Following the last NIR irradiation session, the mice were sacrificed for histology. We could not clearly determine the decrease in size of the irradiated tumors. However, histological examination by Ki-67 immunohistochemical staining showed that cancer cell proliferation was decreased in the base (four tumors) or superficial (two tumors) region of the irradiated tumors compared to nonirradiated tumors (Figure 5c). ssDNA immunohistochemistry, which was used for assessing apoptosis, showed negative staining in all PIT-treated and PIT-untreated tumors. It was reported that PIT causes necrotic cell death, but not apoptosis, on the cell membrane of targeted tumor [28,29]. 

## 3. Discussion

**Figure 6:**
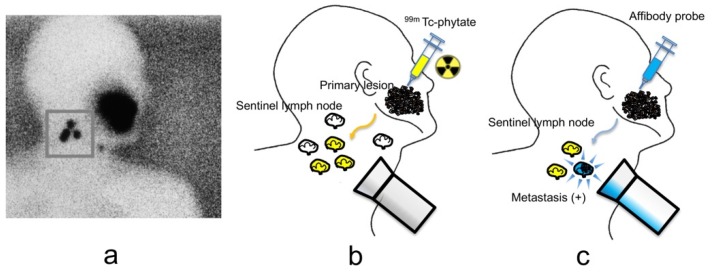
(**a**) Lymphoscintigraphy (^99m^Tc-phytate) in a patient with oral cancer shows increased uptake in sentinel lymph nodes; (**b**) the locations of the sentinel lymph nodes (yellow), even those deeply within the body, could be detected by ^99m^Tc-phytate injection around primary tumor; (**c**) after identification of the sentinel lymph nodes, metastatic cancer cells (blue) in those lymph nodes could be revealed by NIR fluorescence imaging during the biopsy procedure. Metastatic cancer cells may be eradicated by photoimmunotherapy.

In our animal studies, an anti-EGFR affibody probe was able to image metastatic squamous carcinoma cells in lymph nodes. We propose a two-phase combined imaging method. In the first phase, the locations of SLNs, even those deeply within the body, can be detected by ^99m^Tc-phytate lymphoscintigraphy. After identification of the SLNs, NIR fluorescence imaging can be used to reveal metastatic cancer cells in the SLNs during the biopsy procedure (Figure 6). In our animal studies, high-uptake lymph nodes were detected after local injection of the radiotracer. NIR fluorescence imaging was also performed and was able to detect high signals of metastatic cancer cells in the lymph nodes. This strategy could help to spare organs in patients with early-stage lymph node metastasis. In this procedure, ^99m^Tc-phytate was injected after the anti-EGFR affibody probe was injected. Macrophages captured particles larger than 0.5 micrometers. The size of ^99m^Tc-phytate was estimated to be between 200 and 1000 nm. Radiopharmaceuticals accumulated primarily via phagocytosis in macrophages lining the sinusoid spaces in SLNs [2]. In our previous study, TEM revealed that silica imaging nanoparticles approximately 40 nm in size were taken up and localized in phagosomes of macrophages in SLNs [30]. Macrophage accumulation is favorable for detecting SLNs. However, this nonspecific accumulation could cause difficulties in visualizing specific target signals when combined with molecular imaging. Another concern is that the large size of phytate aggregated with serum albumin might interfere with the distribution of affibody probes in lymph nodes if the target probes were injected second. Furthermore, ^99m^Tc decays with a physical half-life of six hours, which restricts the imaging procedure. Thus, we first injected the affibody probes in this combined imaging method.

Imaging probes with a high target-to-background signal ratio are essential for in vivo imaging. Good probe-to-target affinity alone does not guarantee the success of in vivo imaging. If there is high signal concentration in nontarget cells, tissues, or circulating blood surrounding the target, the target cells or tissues become invisible. Frangioni described that the hydrodynamic diameter and charge-to-mass ratio are major determinants of how quickly an agent can extravasate from the vascular space toward malignant cells and how quickly the background signal can be cleared from the body [31].

Intact monoclonal antibodies (mAbs) have long circulating half-lives due to their slow blood elimination. It is considered that conjugating imaging probes with mAbs leads to a low target-to-background ratio, diminishing the image contrast. On the other hand, lower-molecular-weight mAb fragments show shorter half-lives and demonstrate improved tumor penetration and more homogeneous tissue distributions than intact mAbs [32]. In our studies, we used affibodies, i.e., protein-engineered fragments, which have a very low molecular weight (approximately 6.5 vs. 150 kDa of intact mAbs). Affibody molecules are favorable for diagnostic imaging because of their rapid excretion from blood vessels and tissue penetration [21,33]. Although up to now only a few targeted proteins have been available, affibody conjugates with fluorescent dye are used for experimental imaging of glioma [26], HER-2 expressing tumors [34], EGFR-positive thyroid cancer [35], Epidermoid carcinoma [36], etc. Reports of preclinical radionuclide imaging (PET, SPECT) using affibody are well summarized by Krasniqi et al. [27]. Affibody related clinical trials have started in breast cancer, head and neck cancer, soft-tissue sarcoma, glioma, and psoriasis (database of privately and publicly funded clinical studies conducted around the world, ClinicalTrials.gov). The possible capabilities of diagnostic and therapeutic applications of affibody molecules are reviewed in detail by Frejd and Kim [21].

It is supposed that affibody probes have much more rapid lymphatic clearance than other mAbs using probes. Our affibody probes flowed into the lymphatic vascular system. The lymphatic system is different from the blood vascular system because of its one-way, open-ended flow channels, which are not subjected to cardiac pumping forces. However, in the lymphatic circulation, lower-molecular-weight imaging probes may also be beneficial in terms of having a high target-to-background signal ratio, although the concept described above is based on the blood circulation. In the initial lymphatic vasculature, the diameter of the lymphatics ranges from 10 to 60 µm, which is significantly larger than the diameter of arteriovenous capillaries (8 µm). The lymphatics do not have tight junctions between lymphatic endothelial cells. Extracellular fluid, macromolecules, macrophages, lymphocytes, and erythrocytes can directly drain into the initial lymphatics through the lymphatic openings, which have a pore size ranging from several micrometers to 10-20 nm [37]. ^99m^Tc-phytate and our affibody probes could thus enter the lymphatic vessels via these pores.

Many molecular-targeted imaging probes employ mAbs. It is known that the degradation of antibody-drug conjugates occurs by proteolysis via specific and nonspecific mechanisms. Macrophage phagocytosis plays a major role in nonspecific proteolysis [38,39]. Imaging probes conjugated with mAbs would deteriorate in a similar fashion. When the mAb-conjugated substance is subcutaneously injected, its volumes of distribution into tissues would be low due to the molecular size [40]. However, affibodies exhibit greater structural stability and solubility than other antibody mimetics, such as scFv antibody fragments [20]. The lower molecular size of affibodies may provide larger volumes of distribution into tissues and the lymphatics for target delivery.

In our dynamic imaging experiment, metastatic lymph nodes were better differentiated from nonmetastatic lymph nodes at three and six hours after injection of the anti-EGFR affibody probe. It seems likely that the anti-EGFR affibody probe accumulated in the metastatic cancer cells in the lymph nodes for a certain long duration while the probes were gradually washed away from the nonmetastatic lymph nodes. It is obvious that the pharmacokinetics and tissue distribution of molecular-targeted drugs can affect the image profile of the tissue of interest. The dynamic studies indicate that timing to image is significant for differentiating metastasis-positive nodes from metastasis-negative nodes. Further research is necessary to investigate the sensitivity of the method in the detection of metastatic cells/clusters within lymph nodes.

NIR PIT was developed by Kobayashi et al. and is a promising novel type of cancer immunotherapy [28,41,42,43]. Its ongoing clinical trial in recurrent head and neck cancer (phase IIa) is investigating the safety and anticancer activity of various doses and repeated cycles of the experimental treatment in patients with inoperable tumors [44].

PIT uses an antibody-photoabsorber (phthalocyanine dye, IR700) conjugate that binds to a specific cell-surface molecule of cancer cells. When NIR light is applied, the cells swell and burst, causing the cancer cell to die instantly. If PIT can be combined with targeted molecular imaging during SLN biopsy, lymphatic metastasis could be treated with less trauma and a more rational approach to eliminate metastasis in a very early phase. We explored the possibility that the affibody probe conjugated with a photoabsorber could be efficient in PIT. Anti-EGFR affibody conjugated with IR700 was used for this experiment. We observed that the affibody probe could significantly reduce the viability of SAS cells after NIR irradiation. The efficacy of the affibody probe was also examined in the squamous cell carcinoma (SCC) xenograft tumor model in mice. SCC cell proliferation was suppressed in the bottom or superficial part of the xenograft tumors without complete tumor shrinkage. This limited effect may have been because the probe injection was cutaneous. Another possible explanation is that few of the injected probes could reach EGFR-expressing tumor cells because the large part of the injected volume would percolate out of the injection site via vascular flow without accessing tumor cells. Furthermore, in part, our lymph node metastasis model may not be appropriate to assure the efficiency of PIT because the xenografted tumors were too large to simulate early-stage lymph node metastasis. If the probe was administered via afferent lymphatic flow to the metastatic lesion in the lymph node, the effect would have been greater. It has been reported that light dosing schedules can modify the effect of irradiation on necrotic cell death [29]. Further research is required to clarify the best light dosing schedule for treating lymphatic metastasis in this approach. Although our results are confined to the intratumor injection model, our study shows that the affibody probes could specifically target EGFR-expressing tumor cells and induce tumor cell damage. A similar approach using EGFR-specific affibody-phthalocyanine conjugates for PIT in a glioblastoma animal model has been reported. The intravenously injected conjugate enabled the specific imaging of EGFR expression and demonstrated therapeutic efficacy in subcutaneous glioma xenografts [45].

## 4. Materials and Methods

### 4.1. Affibody Fluorescence Imaging Probes

**Figure 7:**
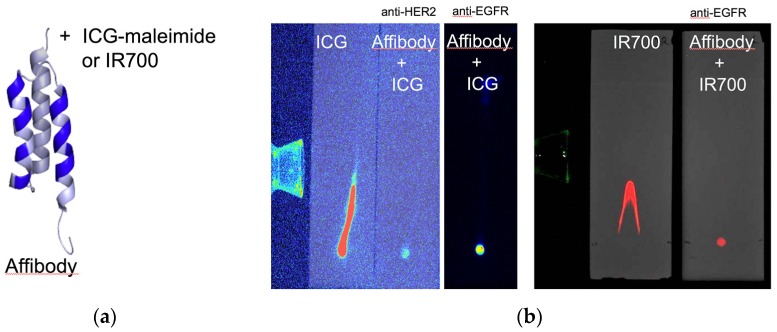
Affibody molecules are shown with randomized positions in the binding site, which are indicated in blue. The molecule was labeled with ICG maleimide or IRDye 700. The figure was adopted from Wallberg H, et al. [46] (**a**). Thin-layer chromatography (TLC) showed that the fluorescence droplet spots of ICG-conjugated anti-EGFR, anti-HER2 affibody, and IR700-conjugated anti-EGFR affibody (2 µL) stayed at the starting point; in contrast, free ICG and free IR700 ran on the slides (**b**).

Anti-HER2 and anti-EGFR affibodies were purchased from Affibody AB, Solna, Sweden. The lyophilized affibodies were dissolved in phosphate-buffered saline (PBS) to a final concentration of 1 mg/mL and then mixed with dithiothreitol (DTT) to a final concentration of 20 mM at >pH 7.5. After incubation at room temperature for 2 h, excess DTT was removed by passage through an NAP5 column (GE Healthcare, Chicago, IL, USA). ICG-maleimide derivative I (1 mg, 50 nmol/tube, Dojindo, Kumamoto, Japan) was dissolved in 1 mL of dimethyl sulfoxide (DMSO). This solution was added to the affibody conjugate and incubated at 37 °C for 60 min, followed by desalting using a protein desalting spin column (Thermo Fisher Scientific, Waltham, MA, USA) and centrifuged at 1500× *g* for 2 min to produce the affibody fluorescence imaging probes (i.e., affibody probe). NIR agent conjugation was confirmed by thin-layer chromatography (TLC) utilizing TLC silica gel 60 F254 (Merck KGaA, Darmstadt, Germany) and 50% ethanol solution (Figure 7a,b).

### 4.2. Cell Culture

EGFR-overexpressing oral squamous carcinoma cells (SAS cells) and breast carcinoma cells expressing low EGFR levels (MCF-7 cells) were obtained from RIKEN BioResource Center, Tsukuba, Japan. SAS cells were cultured in DMEM (GIBCO^®^, Life Technologies, Waltham, MA, USA) supplemented with 10% fetal bovine serum (FBS) and 1% penicillin-streptomycin. MCF-7 cells were cultured in MEM (GIBCO^®^, Life Technologies) supplemented with 10% FBS and 1% penicillin-streptomycin. Both cell lines were maintained in a humidified environment containing 5% CO_2_, T = 37 °C. The medium was changed every other day.

Anti-EGFR affibody ICG probes (1 μL of 70.72 μM affibody) were added to the cultures of SAS and MCF-7 cells (100 μL medium). The cells were then observed using a Zeiss optical microscope (Axiovert 25, Carl Zeiss AG, Oberkochen, Germany) equipped with an NIR filter (ICG BrightLine, Semrock, Inc., Rochester, NY, USA) and a digital camera.

The efficiency of the anti-HER2 affibody fluorescence imaging probes was examined using SK-BR3 (HER2, high expression) and MDA-MB231 (HER2, negative to low expression) cells. The SK-BR3 and MDA-MB231 human breast cancer cells were obtained from the American Type Culture Collection (ATCC^®^, numbers HTB-30™ and HTB-26™). These cell lines were used for comparisons of HER2 expression. The HER2-positive SK-BR3 cells were cultured in McCoy’s 5A medium (GIBCO^®^, Life Technologies) supplemented with 10% FBS (GIBCO^®^, Life Technologies) and 1% penicillin-streptomycin (Invitrogen, Life Technologies, Waltham, MA, USA). MDA-MB231 cells (low HER2 expression) were cultured in DMEM (GIBCO^®^, Life Technologies) supplemented with 10% FBS and 1% penicillin-streptomycin (Invitrogen, Life Technologies). Both cell lines were maintained in a humidified environment containing 5% CO_2_ t 37 °C. The medium was changed every other day. Fluorescence of the anti-HER2 affibody imaging probes was examined as described for the anti-EGFR affibody imaging probe analysis (1 μL of 149.04 μM affibody was added to 100 μL medium).

EGFR and HER2 protein expressions were assessed by immunohistochemistry. Anti-EGFR antibody (clone 31G7, ready-to-use, Nichirei Bioscience Inc. Tokyo, Japan) and anti-HER2 antibodies (primary mouse monoclonal antibody (SV2-61 gamma, Nichirei Bioscience, Tokyo, Japan)) were used as the primary antibodies. Then, the samples were treated with secondary antibodies (Histofine Simple Stain MAX-PO (M), Nichirei Bioscience Inc.) and (Histofine HER2 Kit (MONO) and the Universal Kit (Nichirei Bioscience, Tokyo, Japan)), respectively. The staining procedures were performed according to the manufacturer protocol. Nuclei were stained with hematoxylin.

### 4.3. Analysis of Metastasis Lymph Node Sections from Breast Cancer Patients

#### 4.3.1. Immunohistochemical Examination

To assess the efficiency of the affibody probes, we used deparaffinized sections of lymph nodes from four breast cancer patients; 5-μm-thick sections of both HER2-positive and HER2-negative lymph node metastases were examined. HER2 immunohistochemical staining was performed using the Histofine^®^ HER2 Kit (MONO) and the Universal Kit (Nichirei Bioscience) according to the manufacturer instructions with calibrated positive and negative controls (formalin-fixed, paraffin-embedded, cultured human breast cells: positive control, SK-BR-3 cells; negative control, HeLa cells). HER2 was detected using the primary mouse monoclonal antibody (SV2-61 gamma). The sections were also stained with hematoxylin and eosin (H&E).

#### 4.3.2. Anti-HER2 Affibody Fluorescence Imaging Probe

Sections from the same immunohistochemical studies were immersed in the imaging probe solution for 30 min at room temperature followed by rinsing with PBS twice. The NIR fluorescence of the prepared slides was observed with a Zeiss optical microscope (Axiovert 25, Carl Zeiss AG) equipped with an NIR filter (ICG BrightLine, Semrock, Inc., Rochester, NY, USA) and a digital camera.

### 4.4. Animal Imaging

Five-week-old female athymic mice (SHO, Crlj:SHO-Prkdc^scid^Hr^hr^, Charles River Laboratories Japan, Yokohama, Japan) were used in the in vivo studies.

#### 4.4.1. Metastatic Oral Cancer Mouse Model

Oral cancer was established in SHO mice by injecting 50 µL of SAS cell suspension (1 × 10^7^/mL cells in serum-free DMEM) into the right dorsum of the tongue. The imaging experiments were performed 4–6 weeks after the injection. Lymph nodes in this spontaneous metastasis model were examined by pathology and the cervical metastasis was confirmed by immunohistochemistry.

#### 4.4.2. ^99m^Tc-phytate Lymph Node Imaging

Stannous phytate Kits and a Techne Phytate Kit were purchased from FUJIFILM RI Pharma Co., Tokyo, Japan. ^99m^Tc-phytate solution was prepared by mixing the content of the product with ^99m^Tc-pertechnetate. Using a 27-gauge needle attached to a 1.0-mL syringe, a dose of 9.3 MBq of ^99m^Tc-phytate in 0.1 mL was injected into the right side of the tongue in SHO mice under isoflurane inhalation anesthesia (gas anesthesia system for small animals, DS Pharma Biomedical, Osaka, Japan with Forane^®^, Abbvie, Tokyo, Japan). Two hours later, the mice were euthanized with an overdose of isoflurane inhalation, and the neck tissue was removed. Autoradiography was performed to detect radioactivity in the lymph nodes of the mice using a phosphorimager (FLA2000; FUJIFILM, Tokyo, Japan) with an imaging plate (IP, BAS-SR2040, FUJIFILM, Tokyo, Japan). The excised neck specimens were placed on the IP covered with a sheet of film of polyvinylidene for 5 min to produce autoradiographic images in a darkroom. Then, the IP was processed to image the radioactivity using the FLA2000 device.

#### 4.4.3. NIR Imaging

The anti-EGFR affibody probe (0.05 mL of 70.72 μM affibody) was injected into the tongue surrounding the xenograft tumor. NIR whole-body fluorescence images were captured using an LI-COR Pearl Imager (700 nm channel, LI-COR Biosciences, Lincoln, NE, USA) under isoflurane inhalation anesthesia periodically from 30 min to 24 h after injection. After completion of the NIR whole-animal imaging process, mice were euthanized with an overdose of isoflurane inhalation, and the neck region was dissected for lymph node identification. Excised lymph nodes were also imaged using a NIR imager.

#### 4.4.4. Dynamic NIR Fluorescence Imaging

NIR fluorescence image acquisition was performed using an LI-COR Pearl Imager (700 nm channel, excitation/emission; 685/720 nm) at 30 min and 1, 2, 3, 4, 6, and 24 h after EGFR affibody probe injection into the tongue. Regions of interest (ROIs) were placed on the lymph nodes of tumor-bearing and nontumor-bearing mice, and the intensity of NIR fluorescence was measured as a mean pixel value using the Pearl Cam Software (version 2.0). Node-to-background intensity ratios were calculated by dividing the mean pixel value by the signal intensity in the selected background ROI. The background ROI was placed on a peripheral area surrounding animals. Thereafter, the time-dependent NIR fluorescence intensity ratio of the anti-EGFR affibody probe was represented as a percentage of the initial signal intensity ratio (30 min).

#### 4.4.5. Combined Imaging Method

The combined imaging method based on NIR imaging and ^99m^Tc-phytate lymph node imaging was carried out in mice. For NIR imaging, affibody probe solution was injected into the tongue of SHO mice. Two hours later, ^99m^Tc-phytate solution was injected into the tongue of the mice for lymph node imaging. One hour later, NIR imaging was performed, and the mice were sacrificed with an overdose of isoflurane inhalation.

#### 4.4.6. Immunohistochemical Examination

After NIR imaging of the lymph nodes, the specimens were fixed in 10% formaldehyde solution overnight and embedded in paraffin blocks. They were sectioned at a thickness of 3 µm. Immunohistochemical staining for EGFR in deparaffinized sections was carried out according to the manufacturer protocol. Anti-EGFR antibody (clone 31G7, ready-to-use, Nichirei Bioscience Inc., Tokyo, Japan) was used as the primary antibody. Then, the samples were treated with secondary antibodies (Histofine Simple Stain MAX-PO (M), Nichirei Bioscience Inc.). Nuclei were stained with hematoxylin.

### 4.5. PIT

#### 4.5.1. Affibody PIT Probe

Anti-EGFR affibody was dissolved in PBS to a final concentration of 1 mg/mL and was then added dithiothreitol (DTT) to a final concentration of 20 mM at >pH 7.5. After incubation at room temperature for 2 h, excess DTT was removed from the conjugate by passage through an NAP5 column (GE Healthcare). Three microliters of *N*-(2-Aminopropyl) maleimide (Santa Cruz Biotechnology, Santa Cruz, CA, USA) was then added to the affibody solution. After an hour of incubation at room temperature, 100 µl of the solution was applied to protein desalting spin columns (Thermo Fisher Scientific) and centrifuged at 1500× *g* for 2 min to remove excess *N*-(2-aminopropyl) maleimide. Two microliters of IRDye 700DX (LI-COR, Lincoln, NE) solution (5 mmol/mL in DMSO) was added to 100 µL of the affibody conjugate and incubated at 37 °C for 30 min. Then, the final solution was desalted using protein desalting spin columns (Thermo Fisher Scientific). The conjugation of fluorescent labels of probe was confirmed by thin-layer chromatography before every experiment.

#### 4.5.2. In Vitro PIT

In all, 2 × 10^4^ SAS cells were seeded on 96-well plates and incubated for 24 h at 37 °C and 5% CO_2_. Five microliters of affibody PIT probe solution (70.32 μM affibody) were added to the medium and incubated overnight. After washing with PBS, the cells were irradiated for 40 min with a custom-built illuminator (LED emission: peak wavelength, 690 nm; SMBB690D-1100-02 × 8, EPITEX, Inc., Kyoto, Japan). The power density of each LED was 200 mW/cm^2^ at 500 mA, as measured by a photon detection head (PH100-Si-HA, Gentec Electro-Optics, Inc., Quebec, Canada) attached to a calibrated meter (MAESTRO, Gentec Electro-Optics, Inc., Quebec, Canada). The viability of the irradiated cells was analyzed by Cell Counting Kit 8 (CCK-8, Dojindo Molecular Technologies, Kumamoto, Japan) assay according to the manufacturer protocol. CCK-8 is a convenient assay that shows the proportion of living cells using WST-8 [2-(2-methoxy-4-nitrophenyl)-3-(4-nitrophenyl)-5-(2,4-disulfophenyl)-2H-tetrazolium, monosodium salt].

#### 4.5.3. In Vivo PIT

Five-week-old female SHO mice were used for the PIT experiment. After the mice were acclimatized for one week, SAS cells were xenografted subcutaneously in the back or head of the mice. After 3 to 4 weeks, 0.05 mL of affibody PIT probe solution (70.32 μM affibody) was injected into the established tumors. One hour after the injection, the tumors were irradiated using the custom-built NIR illuminator at 200 mW/cm^2^ and 500 mA 6 times for 10 min/each at an hourly interval. The affibody PIT probe injection and irradiation sessions were performed 3 days a week. As a control, the rest of the tumors were not exposed to NIR irradiation. One hour after the last irradiation, all tumors were removed and fixed with 10% formaldehyde solution. Specimens were embedded in paraffin blocks, and five-µm-thick sections were studied by immunohistochemical staining for EGFR (Envision Kit; Dako, Glostrup, Denmark) using anti-EGFR antibody [31G7, Histofine^®^ Simple Stain™ MAX PO (MULTI), Nichirei, Tokyo, Japan], anti-Ki-67 antibody, and anti-ssDNA antibody as primary antibodies, followed by H&E staining.

### 4.6. Statistical Analysis

For analysis of the dynamic NIR fluorescence imaging of mice, two-way repeated measures ANOVA with the Bonferroni post hoc test was used. One-way ANOVA, followed by Scheffe’s F test, was performed to compare data between groups in the in vitro PIT experiment. Significance was set at *p* < 0.05. Data are expressed as the mean ± standard deviation.

## 5. Conclusions

We demonstrated that combining ^99m^Tc-phytate scintigraphy and anti-EGFR affibody NIR fluorescence imaging can efficiently identify metastatic cancer cells in lymph nodes in real time. Moreover, we demonstrated the theranostic potential of this approach by imaging and treating metastatic cancer cells via PIT. Our study results provide a foundation for the further development of this promising imaging and treatment strategy for earlier lymph node metastasis detection and treatment. Although further research is required, it would guide the path toward translational clinical trials in the future.

## Abbreviations

cRGDY	cyclic arginine-glycine-aspartic acid-tyrosine
CT	computed tomography
DMSO	dimethyl sulfoxide
DTT	dithiothreitol
EGFR	epidermal growth factor receptor
FDA	U.S. Food and Drug Administration
HER2	human epidermal growth factor receptor 2
ICG	indocyanine green
NIR	near-infrared
mAbs	monoclonal antibodies
MRI	magnetic resonance imaging
PBS	phosphate-buffered saline
PEG	polyethylene glycol
PET	positron emission tomography
PIT	photoimmunotherapy
SCC	squamous cell carcinoma
ssDNA	single-stranded deoxyribonucleic acid
SLN	sentinel lymph node
TEM	transmission electron microscopy
TLC	thin-layer chromatography
